# Topical Drug Delivery to the Posterior Segment of the Eye

**DOI:** 10.3390/pharmaceutics14010134

**Published:** 2022-01-06

**Authors:** Marina Löscher, Chiara Seiz, José Hurst, Sven Schnichels

**Affiliations:** Centre for Ophthalmology Tübingen, University Eye Hospital Tübingen, Elfriede-Aulhorn-Str. 7, 72076 Tübingen, Germany; marina.loescher@med.uni-tuebingen.de (M.L.); Chiara.seiz@web.de (C.S.); jose.hurst@med.uni-tuebingen.de (J.H.)

**Keywords:** ocular drug delivery, ocular pharmacokinetics, ocular barriers, permeability, drug characteristics, retinal diseases

## Abstract

Topical drug delivery to the posterior segment of the eye is a very complex challenge. However, topical delivery is highly desired, to achieve an easy-to-use treatment option for retinal diseases. In this review, we focus on the drug characteristics that are relevant to succeed in this challenge. An overview on the ocular barriers that need to be overcome and some relevant animal models to study ocular pharmacokinetics are given. Furthermore, a summary of substances that were able to reach the posterior segment after eye drop application is provided, as well as an outline of investigated delivery systems to improve ocular drug delivery. Some promising results of substances delivered to the retina suggest that topical treatment of retinal diseases might be possible in the future, which warrants further research.

## 1. Introduction

Topical delivery is the safest and easiest method to apply ocular medication, as it can be applied non-invasively by the patients themselves. Nevertheless, the drug absorbance and permeability are low and, as a consequence, the drug concentration in the eye drops is very high and can lead to side effects [1,2].

Diseases of the anterior segment are generally treated with eye drops. Topical treatment of retinal diseases is also desired, as commonly used injections require frequent application by a specialized ophthalmologist and are associated with various side effects. However, topical drug application faces various obstacles, especially if the drug needs to reach the posterior segment. Besides eye drops, topical application via hydrogels, consisting of a network of natural or synthetic polymer chains, is also possible [3]. For glaucoma treatment, the application of hydrogel, in the form of contact lenses, has been investigated. Timolol containing nanoparticles were loaded onto contact lenses and showed timolol release and IOP reduction over 5 days [4]. In situ gels are hydrogels applied as solutions, which can quickly transition from sol-to-gel due to chemical and/or physical crosslinking [5]. The short duration of action, as well as the rapid excretion rate, is often the limiting factor with conventional eye drops. In situ gel systems may provide a potential solution to these problems. However, it is still unclear what influence the in situ gels have on sustained release behavior and tissue distribution in the eye. A recent study on ocular drug delivery of thermosensitive in situ gels, loaded with betaxolol hydrochloride, showed prolonged drug release [6]. Most of the ocular hydrogel research, however, is focusing on intravitreal injections to obtain sustained drug release from a reservoir in the vitreous, and a recent overview was given by Blessing et al. [3]. Another option for achieving prolonged posterior segment delivery are inserts and implants. An overview on new developments was given by Castro-Balado et al. [7]. As those applications still require an invasive procedure, they are not included in this review. Our focus is solely on eye drops as the most common, non-invasive application method.

In this review, we will focus on eye drop medication against glaucoma and age-related macular degeneration (AMD), since those diseases are among the leading causes for blindness worldwide. With increasing age, the prevalence of both diseases rises tremendously [8]. In a large meta-analysis of the last two decades, the prevalence of primary open angle glaucoma ranges from 0.4% at age < 40 to 9.2% at age > 80, with an overall global prevalence of 2.4% [9]. Another large meta-analysis showed the overall prevalence of AMD in the age group of 45–85 to be at 8.69%, ranging from 3.49% at age 45–49 to 24.96% at age 80–85 [10]. Due to the aging population, the number of patients suffering from AMD and glaucoma will reach 288 million [10] and 111.8 million [8] in 2040, respectively.

Glaucoma comprises a group of neurodegenerative diseases, leading to optic nerve damage and retinal ganglion cell death, which ultimately results in vision loss [11,12]. Most forms of glaucoma are associated with an increased intraocular pressure (IOP) [13]. IOP is currently the main line of therapy to slow down the neurodegenerative progression of the disease [14]. Therefore, IOP unrelated therapies are increasingly investigated for the treatment of glaucoma to stop neurodegeneration [15]. 

AMD is a multifactorial disease influenced by genetic disposition, life style factors, and, especially, aging [16,17]. Although the early forms of AMD are typically without symptoms, the two late forms, atrophic and exudative AMD, cause slow (atrophic) or rapid (exudative) vision deterioration and result in severe visual impairment and even legal blindness [18]. In spite of the introduction of the anti-vascular endothelial growth factor (VEGF) therapy, which can slow the progression of exudative AMD, the therapeutic options for AMD are far from satisfactory [19]. No therapeutic options exist to slow or halt the progression from early to late AMD, and treatment possibilities for the early or atrophic forms of AMD are lacking. Improving and prolonging treatment efficacy, as well as reducing the number of injections needed, is the goal of several new therapeutic developments, including targeting additional pathways, combination therapy, and new drug delivery systems [20]. 

In this review, our focus is on non-invasive topical drug delivery. We will first provide an overview of the ocular barriers that a topically applied drug has to overcome to reach the back of the eye, as well as the different absorption pathways that a drug can take (Section 2). Additionally, an insight into the animal models used for ocular drug delivery studies is given and compared to the human morphology (Section 3). Next, several examples of anti-glaucoma and -AMD drugs, which did show successful delivery to the posterior segment after topical application, are presented (Section 4), as well as some promising drug delivery systems that are applied to improve topical delivery to the back of the eye (Section 5). 

## 2. Barriers of Topical Delivery to the Posterior Segment of the Eye and the Influence of Drug Characteristics

**Figure 1:**
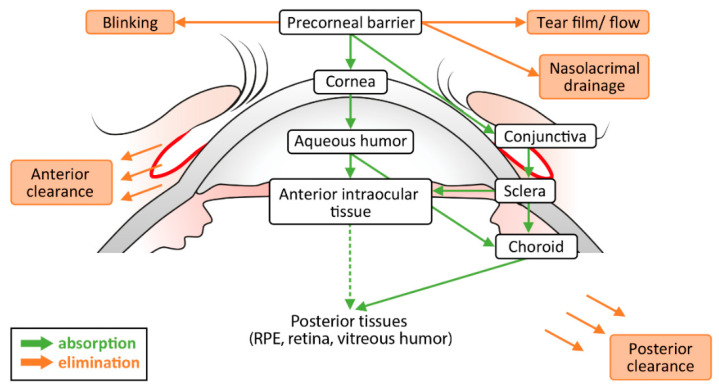
Schematic overview of drug absorption and elimination after topical application.

To reach the posterior segment of the eye, a topically applied medication has to cross various barriers. A schematic overview of drug absorption and elimination is given in Figure 1. Drug penetration is hindered by static, as well as dynamic impediments. Static obstacles include the various layers of the ocular tissues (cornea, conjunctiva, sclera, and retina), as well as the vascular blood-retinal and blood-aqueous barriers [21]. Tear dilution, lymphatic clearance, efflux pumps, and choroidal, as well as conjunctival blood flow, are among the dynamic parameters responsible for impeded drug delivery [22].

### 2.1. Penetration of the Precorneal Layer

The first barrier, which already washes away up to 99% of the active ingredient, is the precorneal layer, the tear film. The tear film consists of three components: the lipid layer (which prevents evaporation), aqueous layer, and underlying mucosal layer (which is composed of a variety of soluble and membrane-bound mucins). Whereby, according to the latest findings, the last two are combined to form an aqueous mucous layer [23]. This tear film, as well as the blinking of the eyelid, effectively prevent the penetration of substances and result in only 1–5% of the applied drug remaining on the ocular surface for a sufficiently long time to become effective there [1]. However, a large part of the flushed out drugs can be reabsorbed by the nasal mucous membrane, which is the reason for systemic side effects [2]. Due to the amphiphilic properties of the tear film, purely hydrophobic, as well as purely hydrophilic substances, have a particularly difficult time penetrating this barrier.

### 2.2. Corneal or Conjunctival Absorption

Absorption into the eye of topically administered drugs may occur through the cornea and/or conjunctival epithelium. Following corneal absorption, drugs may reach the tissues of the posterior segment of the eye by passing through the anterior segment. After drug absorption into the conjunctiva, the vitreous and retina can be reached, either by diffusion through the sclera or cornea or via clearance into the systemic circulation [21].

At physiological pH the cornea behaves as a negatively-charged membrane, therefore positively-charged molecules penetrate more easily than negatively-charged compounds [24]. The multilayered structure of the cornea further complicates the uptake of active substances. The epithelial cell layer has lipophilic properties and is generally the rate-limiting barrier to transcorneal transport. The underlying stroma is hydrophilic and the endothelial layer below is lipophilic [25]. Lipophilic drugs take the transcellular route through the corneal epithelium, whereas hydrophilic compounds cross the epithelial barrier via the paracellular pathway. Paracellular permeation is limited by the paracellular pore diameter. The corneal epithelium only allows hydrophilic compounds with a size < 500 Da to permeate due to its paracellular pore diameter of 2.0 nm ± 0.2. Whereas the conjunctiva allows permeation of molecules of size 5–10 kDa, due to its paracellular pore size of 3.0 nm ± 1.6 [26] (Table 1).

Absorption through the cornea and conjunctiva is easier for lipophilic drugs. The lipophilic drug propranolol, for example, is absorbed 5–10-fold greater than sotalol, a hydrophilic drug with the same molecular weight [27]. Lipophilic compounds with lipid/water partition coefficients (LogD values) of 2–3 are considered optimal for corneal permeation. Compounds with even higher values (logD > 3) show lower permeability, due to slower desorption from the lipophilic epithelium to the hydrophilic stroma. Therefore, different lipophilicity is not the determining factor for the corneal permeability of highly lipophilic pilocarpine prodrugs, but rather the conversion rate of the prodrug to the more hydrophilic parent drug, which allows an easier transfer from the epithelium to the stroma [28]. 

Besides passive diffusion, active transport also plays a role in drug penetration of the cornea. As hydrophilic drugs only show low passive diffusion across the cornea, the impact of active transport may be more pronounced, compared to lipophilic drugs. For lipophilic drugs, the transfer from the epithelium to the stroma is the limiting step; therefore, the efflux transport in the basolateral side of the epithelium has a higher impact. Nevertheless, since drug absorption via transporters is saturable, passive diffusion is possibly the dominating mechanism [29].

Even though the conjunctiva is more permeable than the cornea, the presence of efflux pumps impedes substance transport via this pathway. In addition, the existing vascularization of the conjunctiva, as well as the episcleral, leads to a removal via the systemic circulation. The bioavailability of drugs in the anterior chamber is, therefore, at best 5% [30]. Several macromolecule transporters (for amino acids, nucleosides, d-glucose, monocarboxylate, and dipeptides) are expressed in the conjunctiva that may be relevant for ocular drug delivery. The amino acid transporter ATB^0,+^, for example, recognizes almost all amino acids, making it a feasible target for amino acid derivatives (including prodrugs) [31].

### 2.3. Permeation through the Intraocular Tissues

After crossing the cornea and reaching the aqueous humor, the drug diffuses to the surrounding intraocular tissues and vitreous humor [32]. Further barriers for drug permeation are the removal via the aqueous humor and the intracellular degradation of active substances by metabolizing enzymes, such as glutathione [33]. Diffusion across the vitreous is easier for small molecules [34]. However, as the mesh size of the vitreous is estimated at 500 nm, size is not the limiting factor for particle diffusion [35]. The influence of charge on particle diffusion is much more pronounced [36]. Since negatively-charged hyaluronic acid and glycosaminoglycan proteins exist in the vitreous body, negatively-charged particles, such as polylactic co-glycolic acid (PLGA) or human serum albumin, diffuse better than cationic particles [35,37,38]. To improve the migration of positively-charged particles through the vitreous, attempts are being made to coat them with polymers, such as polyethylene glycol (PEG) and hyaluronic acid [39,40,41]. Another dominating factor influencing the passage through the vitreous body and, consequently, the intraocular half-life is the lipophilic properties of a substance. Hydrophilic and large compounds remain in the vitreous for a longer time and are typically removed via anterior elimination [42]. Whereas small and lipophilic molecules mainly take the posterior route, as they can easily cross the retina [43]. Pitkänen et al. discovered that lipophilic β-blockers passed the outer blood-retinal-barrier much more efficiently than more hydrophilic β-blockers of similar size [44].

Besides the transcorneal diffusion through the anterior chamber to the vitreous and retina, topically applied drugs can also enter the systemic circulation and reach the retina via retinal vasculatures [45]. This periocular drug absorption includes diffusion of the drug through the conjunctiva to the Tenon’s capsule and, further, through the sclera and choroid to the retina. Although most of the administered dose is removed into the systemic circulation [46] (Figure 1).

Scleral permeability is strongly dependent on molecular weight and radius. Smaller molecules are more permeable than bigger ones. The sclera can be crossed by molecules up to a size of roughly 70 kDa [47]. Similarly, globular proteins have a better permeability than linear dextrans of the same molecular weight. [48]. Some lipophilic drugs can enter the posterior segment directly by lateral scleral diffusion and subsequently penetrate Bruch’s membrane and the RPE [46]. Hydrophilic compounds can easily penetrate the sclera, as it consists of porous spaces within a collagen aqueous network [48]. At physiological pH the matrix structure of proteoglycans is negatively charged, which facilitates the permeation of negatively-charged compounds through the sclera [49]. For hydrophilic compounds taking the transscleral route, the RPE is most likely the rate-limiting factor. Whereas RPE-choroid and sclera represent comparable barriers for lipophilic drugs [44] (Table 1).

Melanin binding can also effect drug distribution and lead to increased drug concentrations in RPE and choroid. This may, on one hand, reduce receptor binding of the drug; however, on the other hand, melanin binding could prolong drug effects, due to sustained drug release from the melanin depot [50,51].

## 3. Model Systems to Study Drug Delivery to the Retina

To study the absorption of new drugs or delivery systems, different animal models are used. The goal of those preclinical investigations is to predict the clinical performance of the drug candidates. To this end, the characteristics of the model systems have to be carefully considered and compared to the human situation.

Choosing the suitable preclinical model is of utmost importance, as shown by repeated failures of topically applied drugs in clinical investigations, in spite of promising preclinical data in rodents [52]. Animal models used in ocular drug delivery studies include mice, rats, rabbits, monkeys, and sometimes dogs and pigs [45]. There are various characteristics that can be taken into account. The bioavailability of topically applied drugs at the back of the eye can be influenced, among other factors, by differences in the thickness of cornea and sclera, axial length, and vitreous volume. The human vitreous volume as an example is one thousand times larger than in rodents, which may have a strong influence on the intraocular concentration of small molecule drugs applied via eye drops [45].

In the following section, we will outline only a few important parameters and compare the eyes of mice, rabbits, and pigs to the human eye (Table 2).

Mice are the most commonly used species, presumably owing to cost effectiveness, availability of genetically modified strains, and short reproduction cycles [75,76]. However, some data indicate that the small eyes of rodents are not well suited to predict the clinical efficacy of topical drugs [77,78].

Mice eyes have a central corneal thickness of 123–134µm, which is one-fifth to one-fourth of the human cornea (548 µm). The anterior chamber is 0.1 µm deep (factor 30 shorter than the human anterior chamber) and has a volume of around 2.5 µL (little more than 1 percent of the human anterior chamber volume). The difference in vitreous volume is even more pronounced, with the vitreous volume in mice being factor 1000 smaller than in humans (4.4 µL vs. 4400 µL). The cone-based performance in the mouse retina is also not representative for the mammalian retina, with a rod-to-cone ratio of only 97:3 [79,80]. Furthermore, the mouse retina is devoid of a macula or similar retinal region, with a high density of cones, retinal ganglion, and bipolar cells [81].

Ocular pharmacokinetic studies are mostly done in rabbits. Compared to the small rodents, the rabbit eye is much closer to the human situation. Corneal thickness is 349 µm (compared to 548 µm) and, although the anterior chamber depth is only 0.16 µm (compared to 3.05 µm), the anterior chamber volume is more comparable (250 vs. 170 µL). The vitreous volume in rabbit is one-third of the human vitreous (1400 vs. 4400 µL). The retinal thickness is in the same range (at least in the vascular area). While some authors claim the rabbit is a poor model of the human eye, due to the differences in vitreous volumes and vitreous diffusional path length [82,83], others state that for intravitreal pharmacokinetics the rabbit model is clinically predictable, as intravitreal distribution and clearance is quite comparable between rabbits and humans [84]. Rabbits are known to have a visual streak (VS), in which the density of rod and cone photoreceptors, retinal ganglion, and amacrine cells is highest [85]. The neural retina of the rabbit is rather hypoxic and, as only a small area shows any retinal circulation, mainly dependent on choroidal circulation [86]. In contrast to the human eye, optic nerve fibers in the rabbit are already myelinated in the retina [87].

Besides studies in non-human primates, which come with a lot of ethical and financial concerns, the porcine eye resembles the human situation quite well. Regarding morphology, size, and vascularization, human and porcine eyes are comparable [88,89]. The central corneal thickness is in the same range (543–797 µm in pigs vs. 548 ± 35 µm in humans). The anterior chamber depth is one-half to two-thirds, compared to humans (1.77 ± 0.27 vs. 3.05), whereas the anterior chamber volume is roughly 50% bigger, 260 vs. 170 µL. The vitreous volume is 3300 µL vs. 4400 µL in humans. The retinae of humans and pigs are quite comparable, 300 µm thickness in pig vs. 310 µm thickness in humans [73]. Further, the porcine retina is a suitable model for retinal research, as the photoreceptor mosaic of pigs and humans is quite similar. The porcine retina is well provided with cones, an expansive vascular tree, and an area sufficiently devoid of blood vessels, to suggest an *area centralis* near the posterior pole [54]. To study human retinal diseases and drug delivery to the retina porcine (and similarly bovine) eyes and retinal explants are increasingly applied [90,91]. Developing ex vivo models from these waste products of the food industry offers the chance to overcome shortcomings of the currently used in vivo models, while, at the same time, reducing the number of animal experiments [73,90]. Peynshaert et al., for example, recently developed a bovine retinal explant model with an intact vitreoretinal interface, where retinal penetration, following intravitreal injection, can be studied [91,92].

## 4. Retinal Delivery of Different Medical Compounds

As mentioned in the previous chapters, drug delivery to the retina after topical administration faces various barriers. Nevertheless, a certain variety of compounds have shown successful topical delivery to the posterior segment in preclinical models, and some have already been investigated in clinical trials (Table 3).

Pharmacokinetics and distribution can be influenced by different size of the drugs and pH. Corneal penetration can be enhanced by increasing the lipophilicity of the compound. Prodrugs like Latanoprost and Travoprost take advantage of this effect. They contain ester groups, which increases their lipophilicity and, thereby, the uptake into the cornea, where the prodrugs are then metabolized into the active drugs by esterase enzymes [93]. Compared to small molecules, biologics face greater hurdles in topical absorption due to their size. However, they also require only a lower target concentration, as they exhibit higher affinities for their target [52].

### 4.1. Anti-Glaucoma Drugs

Several glaucoma drugs have shown to reach the back of the eye. Dorzolamide hydrochloride was topically applied in Japanese white rabbits. After only 15 min, the drug concentration in the anterior segment of the eye and retina increased significantly and peaked within an hour. This indicates efficient migration of the drug between ocular tissues. It can, therefore, be assumed that carbonate anhydrase activity is immediately suppressed by dorzolamide and that the drug is rapidly distributed in the ocular tract after local administration. [94]. Topical delivery of brimonidine to rabbits and monkeys yielded retinal drug levels sufficient to activate alpha2-adrenergic receptors [95]. Similarly, Betaxolol could be delivered to the retina of patients with glaucoma and cynomolgus monkeys [96].

Netarsudil, a Rho-associated protein kinase inhibitor, was developed as novel treatment option for glaucoma. In preclinical studies, large intra ocular pressure (IOP) reductions were obtained in rabbits and monkeys, and a favorable pharmacokinetic profile was shown. In distribution studies in rabbits with a single topical dose (35 µL) of 0.02% 14C-netarsudil, a Cmax of radioactivity of 80 (left eye) and 50 (right eye) ng ∗ eq/g was reached in the retina [97]. In December 2017, after completing various clinical trials, Netarsudil was approved by the FDA for the treatment of open-angle glaucoma or ocular hypertension [108].

Memantine HCL, an antagonist to NMDA-receptors used for the treatment of Alzheimer’s disease, has neuroprotective properties and might be beneficial in the treatment of glaucoma. In an arterially perfused bovine eye model, memantine was observed to accumulate in the posterior segment. Koeberle et al. hypothesized that melanin-binding may support sustaining significant concentrations in the retina [98].

### 4.2. Anti-AMD Drugs

The greatest desire in ophthalmic drug-delivery development is to identify a topical treatment option for retinal diseases like age-related macular degeneration (AMD). Currently, anti-VEGF antibodies are applied intravitreally to inhibit choroidal neovascularization in patients with AMD [109]. Because of their molecular weight of roughly 150 kDa, topical delivery is highly challenging. After topical application of the VEGF-A inhibitor bevacizumab in pigmented rabbits (1.25 mg/0.05 mL six times daily for the first 7 days), only a small level of bevacizumab was detected in the iris/ciliary body and retina/choroid, not sufficient for a therapeutical effect [100]. In contrast to that, the anti-TNF-alpha single-chain antibody fragment ESBA105, with a molecular weight of only 26 kDa, was distributed to all ocular tissues, following topical application, reaching a retinal concentration of 214 ng/mL after single application and 917 ng/mL after multi-day treatment in rabbits. Systemic drug exposure was reported to be very low [101].

Besides antibodies and antibody fragments, several innovative small molecules and inhibitors of receptor tyrosine kinases have been investigated for their posterior segment delivery.

#### 4.2.1. Innovative Small Molecules

Several small molecule eye drops have shown to reach the posterior segment and were able to develop their effect against choroidal neovascularization (CNV) in preclinical models. In the following section, we outline the promising candidates that were further investigated in clinical trials.

One promising approach is targeting misfolded Amyloid β aggregation, to prevent their neurotoxic effect. Russ et al. have shown that a single topical delivery of GAL-101, a small molecule inhibitor of Aβ, sustained concentrations >100 nm in the retina of monkeys for >2 h. Daily eye drops in a glaucoma rat model achieved >90% neuroprotection [102]. Phase I trials for geographic atrophy have successfully been completed [110].

Recent investigations have demonstrated promising results for drugs targeting arginyl-glycyl-apartic acid (RGD)-binding integrins in ocular tissues [111]. One of those drugs is the integrin αVβ3 antagonist SF-0166 (OcuTerra Therapeutics, Boston, MA, USA; formerly SciFluor Life Sciences, Inc., Boston, MA, USA). Preclinical investigations in rabbits showed a good pharmacokinetic profile, and the efficacy results of topical administration in laser-induced and VEGF-induced CNV rabbit models were comparable to a bevacizumab injection [103]. Furthermore, biological effects and good tolerability have been shown in early clinical trials with diabetic macular edema [112] and neovascular AMD patients, which underlines the potential of targeting RGD-binding integrins, to develop a next-generation therapy for retinal diseases [111].

Most of the investigated therapeutical approaches, however, failed to confirm a positive preclinical outcome. Squalamine lactate, a very promising inhibitor of angiogenesis by a novel intracellular mechanism, was able to reduce choroidal neovascularization in a laser-injury model in the rat when applied intravenously [104]. An eye drop formulation of 0.2% squalamine lactate (OHR-102) was later launched by Ohr Pharmaceutical Inc. (New York, NY, USA). In a Phase II trial (NCT01678963), this formulation, with improved trans-scleral permeability and increased choroidal retention, showed a trend towards better visual acuity in patients with all types of naïve neovascular lesions. Subsequent Phase III trials (NCT02727881) failed to meet the primary endpoint [110].

#### 4.2.2. Inhibitors of Receptor Tyrosine Kinases

TG100801 is an inactive prodrug that generates TG100572 by de-esterification, which inhibits Src kinases and selected receptor tyrosine kinases. Topical TG100801 significantly suppressed laser-induced CNV in mice and reduced fluorescein leakage from the vasculature and retinal thickening, measured by optical coherence tomography in a rat model of retinal vein occlusion [105]. TG100801 could not demonstrate efficacy when investigated in AMD patients (ClinicalTrials. gov identifier: NCT00509548).

Pazopanib is a tyrosine kinase inhibitor that inhibits angiogenesis. To test the inhibitory effect of pazopanib on experimental choroidal neovascularization (CNV), CNV was induced in rats by laser coagulation of Bruch’s membrane. Twice daily topical treatment with pazopanib significantly (*p* < 0.001) reduced leakage from photocoagulated lesions by 89.5% and significantly reduced the thickness of the resulting CNV lesions by 71.7% (*p* < 0.001). In addition, VEGF immunoreactivity was decreased, compared with control eyes [77]. When tested in humans, the compound attained to improve best-corrected visual acuity in a Phase II trial, including patients with sub-foveal CNV secondary to AMD [113]. In a subsequent Phase IIb trial, pazopanib did not show additional benefit, compared to ranibizumab injections and, thereby, failed to meet its primary endpoint [114].

Acrizanib, a VEGFR2 inhibitor, showed impressive in vivo efficacy in the mouse CNV model, by leading to complete inhibition of neovascularization. Three times daily administration of 4 μL × 1.0% suspension/eye in the rat CNV model also resulted in 90% inhibition of neovascular area [106]. When investigated in a clinical trial Acrizanib failed to demonstrate efficacy, compared to anti-VEGF injections [115].

Another VEGFR2 inhibitor, which reaches the retina and choroid via the trans-scleral route, is PAN-90806, by the company PanOptica, Inc. (Mount Arlington, NJ, USA). Preclinical studies demonstrated sustained drug levels in choroid and retina, as well as the suppression of the formation of new abnormal blood vessels. In the Phase I/II trials, the PAN-90806 eye drops were applied as monotherapy in patients with neovascular AMD (once daily for 12 weeks), 51% of which did not need a rescue injection during trial or one month post-treatment [107]. Further clinical investigation is needed to confirm this data.

There will be many reasons for the failure in clinical investigations, but the lack of sufficient investigations in a larger species might be one of them. As has been shown for regorafenib and pazopanib, the drug concentrations in the choroid and retina, after topical application in rabbits and monkeys, were much lower than those in rats and, therefore, not sufficient to inhibit angiogenesis [116].

In interpreting efficacy and distribution data, it has to be taken into account that systemic distribution to the posterior segment, following topical drug application, may vary due to blood volume of the investigated species. Considering the smaller blood volume of rabbits vs. humans (~0.12 l vs. ~5 l), drug levels in the posterior tissues may reach values that cannot be transferred to humans. Therefore, Rodrigues et al. advised to take drug levels and/or efficacy data in the untreated contralateral eye into account when evaluating drug distribution [52].

From these examples, showing promising preclinical data but often failing in clinical studies, it is evident that further improvements are needed. Delivery systems, such as nanoparticles, offer the chance to enhance uptake and permeation of drugs to get a sufficient amount of drug to the retina to be effective there.

## 5. Delivery Systems and Formulation Approaches to Improve Topical Delivery to the Retina

**Figure 2:**
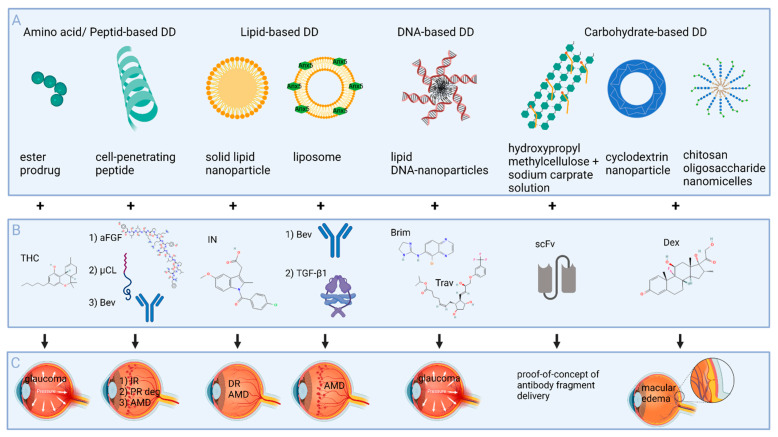
Schematic presentation of drug delivery (DD) systems and formulation approaches to improve topical delivery to the back of the eye. (**A**) Drug delivery systems presented in this review. (**B**) Drugs that were transported by those delivery systems. (**C**) Diseases that are addressed with the DD system and drug. The molecular structures were obtained from https://pubchem.ncbi.nlm.nih.gov (accessed on 21 December 2021). Abbreviations: THC—tetrahydrocannabinol; aFGF—acidic fibroblast growth factor; µCL—calpain inhibitory peptide; Bev—bevacizumab; IR—retinal ischemia/reperfusion; PR deg—photoreceptor degeneration; AMD—age-related macular degeneration; IN—indomethacin; DR—diabetic retinopathy; TGF-β1—transforming growth factor beta 1; Brim—brimonidine; Trav—travoprost; scFv—single-chain variable-domain fragment; Dex—dexamethasone. Created with BioRender.com (accessed on 21 December 2021).

Numerous drug-delivery systems have been investigated, in order to achieve effective drug concentrations in the posterior segment of the eye by topical application (Table 4). Nanoparticles are increasingly applied as drug-delivery systems, on one hand, to enhance the bioavailability of drugs, by increasing their absorption or facilitating their passage through the tissue and, on the other hand, to achieve controlled release of the drug [117]. Polymeric materials have great potential as NP precursors, since their properties can easily be tailored through derivatization of biopolymers or preparation of synthetic polymers, according to drug delivery needs [118]. Drug uptake can also be improved via formulation development approaches, such as the addition of enhancers of viscosity and permeability, as well as prodrug design [119]. In the following chapter, different drug delivery and formulation approaches, based on amino acids/peptides, lipids, DNA, and carbohydrates, will be presented. Figure 2 offers an overview of the delivery systems presented in Section 5, including the drugs that are transported and ocular diseases that are addressed.

### 5.1. Amino Acid/Peptid-Based Drug Delivery

To overcome the anterior segment of the eye, there are many formulation approaches. Recently, cannabinoids, such as tetrahydrocannabinol (THC), have been applied as anti-glaucoma drugs for their IOP lowering effect. However, THC eye drops have poor ability to cross the cornea, due to their high logP value (6.42) and low aqueous solubility (1–2 µg/mL). To overcome this low bioavailability, a valine-hemisuccinate (Val-HS) ester prodrug has been developed. THC-Val-HS achieved significantly higher transcorneal permeability, mainly due to its larger polar surface area, relatively lower logD 7.4, and increased aqueous solubility. Adelli et al. showed significantly higher THC concentrations in the anterior segment of the eye by THC-Val-HS-loaded topical eye drops in anesthetized rabbits. Compared with marketed pilocarpine HCl and timolol maleate eye drops, the intraocular pressure-lowering effect of THC-Val-HS was equivalent to that of pilocarpine [120]. In further development efforts, the prodrug was formulated in a nanoemulsion (NE), which led to a prolonged IOP lowering effect. In normotensive rabbits, the THC-Val-HS-NE showed a better effect than commercial timolol or latanoprost [134].

Another formulation approach for the topical delivery of proteins and peptides are cell-penetrating peptides (CPPs). Herein, using the CPP HIV transactivator of transcription (TAT), Wang et al. delivered acidic fibroblast growth factor (FGF) to rat retina after a single topical administration (2 µg in a 40 µL solution). TAT-aFGF-His proteins were detectable in the retina for at least eight hours and mediated strong protection against ischemia-reperfusion injury compared to the aFGF-His and PBS treated groups: the inner retinal layer structure was better maintained, retinal ganglion cell apoptosis was reduced, and retinal function improved [122].

Ozaki et al. topically delivered a calpain inhibitory peptide (which protects photoreceptors in retinal dystrophic rats) conjugated with TAT to the posterior segment of the rat eye. Application of 20 μL of 1 mM Tat-μCL twice daily for seven days yielded a concentration of 15.3 pg/µg protein one hour after the final instillation [123].

Cogan et al. also succeeded in achieving therapeutic concentrations in the posterior segment of the rat eye by combining bevacizumab with polyarginine-6, another CPP. After a single 20 µL eye drop of bevacizumab (25 µg/µL), a maximum concentration of 1.65 ± 0.26 µg/mL was detected in the retina at 40 min after application. In ex vivo studies on porcine eyes, a single 20 µL eye drop of bevacizumab (25 µg/µL), complexed with CPP, yielded a concentration of 10.68 ± 3.57 µg/mL in the vitreous and 0.10 ± 0.03 µg per retina, which is within the therapeutic range for humans (10–200 µg/mL) [124].

### 5.2. Lipid-Based Drug Delivery Systems

Davis et al. detected physiologically relevant concentrations of bevacizumab in the posterior segment of the eye in rats and rabbits. Here, the antibody was delivered using liposomes functionalized with the anionic protein Annexin A5. A single 0.03 mL dose (containing 13 mg/mL Avastin) yielded a bevacizumab concentration of 127 ng/g in the posterior eye of rats. Application of 0.03 mL eye drops (25 mg/mL Avastin) once per day for five days resulted in 18 ng/g in the rabbit retina/choroid [126]. The comparison of those results to bevacizumab concentrations during clinical treatment with intravitreal injections revealed that the topical liposomal delivery resulted in 3–5 orders of magnitude lower bevacizumab concentrations. Which underlines that major improvements are necessary to achieve clinically relevant results [135].

An Annexin 5 complemented liposome formulation was also used by Platania et al. to deliver growth factor beta 1 topically to the vitreous of rabbits. After topical application (two times within five minutes) of 30 µL eye drops (TGF-ß1 concentration of 125 ng/mL), a maximum concentration of 114.7 ± 12.40 pg/mL was delivered to the vitreous (tmax 60 min) [127].

For the delivery of lipophilic drugs, Balguri et al. have designed various solid lipid nanoparticles (SLN) and nanostructured lipid carriers (NLC), where liquid lipids are incorporated in the solid lipid structure. The delivery of the non-steroidal, anti-inflammatory drug indomethacin (IN) was investigated in albino rabbits. Two doses of 50 µL eye drops to conscious rabbits yielded retinal-choroidal IN-concentrations of 227 ng/g with IN-SLN und 893 ng/g with IN-NLC [128].

### 5.3. Lipid DNA-Based Nanoparticles

Lipid DNA nanoparticles (NPs) are made of alkyl-modified oligonucleotides that represent amphiphilic molecules. Due to microphase separation, these NPs self-assemble into micelles in an aqueous environment. The hydrophobic part (the lipid modifications) forms the core, while the hydrophilic DNA sticks out of the micelle [136,137]. The NPs exhibited relatively low critical micelle concentrations, demonstrating their stability in aqueous surroundings [138]. NPs composed of amphiphilic DNA strands, that were composed from twelve nucleotides, whereof four were lipid-modified with an alkyl chain, adhered best to the ocular surface among different lipid NPs [138]. Brimonidine loading to these lipid-modified DNA-NPs via hydrophobic interactions or using specific aptamers caused improved affinity to the cornea. Maintaining drug release from the NPs. Brimonidine-NPs significantly reduced intraocular pressure in live animals, more than pure brimonidine [129]. With the same delivery system, aptameric loading of travoprost was also achieved. Travoprost delivery with this NP system resulted in longer adhesion to the corneal surface, enhanced uptake, efficacy, and biocompatibility. For example, after four hours, the amount delivered in the eyes of albino rats via these NPs was four times higher, compared to pristine travoprost [130].

### 5.4. Carbohydrate-Based Drug Delivery Systems

A 28-kDa antibody fragment (in a solution supplemented with penetration and viscosity enhancers) could be delivered to the vitreous of rabbits, at concentrations of 50–150 ng/mL 12 h, after topical administration of 50 µL eye drops (at 20 min intervals over 12 h) containing 0.8–1.1 mg/mL protein. The antibody fragment was applied in a solution with sodium carprate as penetration enhancer and hydroxypropyl methylcellulose as viscosity enhancer [131].

γ-cyclodextrin-based (CD) nanoparticles have been developed, by the company Oculis Switzerland (Lausanne, Switzerland), for the treatment of anterior and posterior segment diseases. The cyclodextrins increase the solubility of lipophilic drugs and can enhance topical drug uptake, through constantly supplying dissolved drug molecules to the membrane surface [139]. Multiday application of the anti-inflammatory corticosteroid dexamethasone-loaded CD nanoparticles yielded high concentration in the retina of rabbits (201 ng/g in the treated eye) [132]. In a clinical trial with DME patients, this formulation could significantly improve visual acuity and result in decreased macular thickness, comparable to a posterior subtenon injection of triamcinolone acetonide, a frequently-reported, off-label treatment for DME [140]. Several other clinical studies using γCD eye drops have been conducted, and the development is ongoing to find better treatment options for DME and postcataract surgery inflammation [141].

Xu et al. reported the development of Chitosan oligosaccharide-valylvaline-stearic acid (CSO-VV-SA) nanomicelles. CSO is responsible for increased retention at the ocular surface, and VV is known to target the peptide transporter-1 (PepT-1), which can enhance ocular uptake and penetration of conjunctival, as well as scleral tissue. In an in vivo study in rabbits, therapeutic concentrations of dexamethasone were reached in retina-choroid-sclera via CSO-VV-SA eye drop application [133].

## 6. Conclusions and Future Perspectives

Treating retinal diseases by applying simple eye drops is an attractive goal for ophthalmologists, patients, and the pharmaceutical industry likewise. The biggest challenge in reaching this goal is to achieve sufficient drug bioavailability, while minimizing side effects. Topical treatment of the posterior segment of the eye presents many anatomic and physiologic hurdles. As a result, drug delivery via the eye is very complex. In many cases, in vivo animal models are the most appropriate model to study the absorption of new drugs or delivery systems and to best predict their clinical performance. However, novel ex vivo and in silico models show compatible outcomes [73,142]. It is important that model systems are carefully chosen, evaluated, and compared to the human situation.

Detailed physicochemical characterization of the compound and delivery system is required to predict and evaluate ocular bioavailability and optimize the particle properties accordingly. Additionally, production methods are becoming more and more elaborate, with the goal to produce defined delivery systems, according to drug loading, size, shape, and properties of the drug delivery systems. It is important to incorporate the requirements for large scale production early in the development process to make sure the particles are ready to be produced for later clinical investigations.

As topically delivered drugs take a long journey through the eye to reach the posterior segment and different routes are possible, in the future, targeted delivery, for example, by using aptamers, might be of importance to make sure the target cells/tissues are reached and side effects are minimized. One way to go might be delivery systems comprising of receptor targeting. Additionally, the development of innovative, new small molecule therapeutics would be desirable, as the currently-applied antibody therapeutics are not suitable for topical delivery, due to their size. Safety profiles of the particles are another hurdle in bringing those new technologies from bench to bedside. The focus should lie on the development of biocompatible and biodegradable drug delivery systems with safe degradation products.

Some promising preclinical studies, in which effective drug concentrations were achieved in the posterior segment of the eye by topical application, were highlighted in this review, suggesting that topical treatment of retinal diseases may be possible. However, clinical trials showed that innovative drug-tailored delivery systems are needed for an efficient retinal drug delivery.

In conclusion, innovative delivery systems and more clinical data are needed to sufficiently understand and tailor retinal drug delivery via topical application.

## Figures and Tables

**Table 1:** Influence of drug characteristics on transport of topically applied drugs across ocular tissues.

Ocular Tissues	Size and Radius of the Drug	Charge of the Drug	Drug Characteristics	
			Lipophilic	Hydrophilic
Cornea	<500 Da	Easier penetration of positively-charged molecules	Transcellular, 5–10× greater absorption than hydrophilic drugs, transfer from epithelium to stroma -> rate-limiting	Paracellular <500 Da Low passive diffusion
Conjunctiva	5–10 kDa		Easier than for hydrophilic compounds	Mainly through conjunctiva (9–17 times larger surface area than cornea)
Sclera	<70 kDa, better permeability of globular proteins vs. linear dextrans	Passage of negatively-charged solutes is facilitated	RPE-choroid and sclera are equal barriers	Easier penetration than lipophilic compounds, RPE is rate-limiting
Vitreous	<500 nm Easier diffusion of small molecules	Negatively-charged particles diffuse better than cationic particles	Easier than for hydrophilic compounds	Longer half-life

**Table 2:** Ocular characteristics of commonly used preclinical models vs. the human eye (modified and supplemented according to Wang et al. [45]).

Parameter	Mice	Rabbit	Pig	Human
Blink intervals [blinks per minute]	300 [53]	360 [53]	10 [54]	5 [53]
Central corneal thickness [µm]	123–134 [55]	349–384 [56]	543–797 [57]	548 ± 35 [58]
Anterior chamber depth/ocular axis [mm]	0.1 [45]	0.16 [45]	1.77 ± 0.27 [59]	3.05 [45]
Anterior chamber volume [µL]	2.39–3.08 [60]	~250 [45]	~260 [61]	~170 [62]
Aqueous humor production [µL/min]	0.18 ± 0.05 [63]	1.46 ± 1.71 [64]	3–4 * [65]	2.4 ± 0.6 [66]
Vitreous volume [µL]	4.4 ± 0.7 [67]	~1400 [68]	3300 [69]	~4400 [70]
Mean Retinal thickness [µm]	204 [71]	Vascular area 163–340, avascular area 142–168 [72]	300 [73]	310 [73]
Average RGC density [cells/mm^2^]	4000 [74]		6000 [74]	5700 [74]

* aqueous flow rate.

**Table 3:** Overview of topically applied drugs reaching the posterior segment in preclinical investigations.

Compound	Characteristics	Size	Physiological Charge	logP *	Preclinical Investigations	Cmax Retina	Ref.
Drugs tested for glaucoma treatment							
Dorzolamide (hydrochloride)	inhibitor of carbonic anhydrase	324.4 Da (360.9 Da)	1	Dorzolamide: −0.15	Japanese white rabbits: 1 drop of 1% dorzolamide hydrochloride eyedrops -> Cmax after 1 h	3.79 µg/g	[94]
Brimonidine	Alpha2-adrenergic agonist	292.13 Da	1	1.37	Monkeys: 14 days 0.5% brimonidine twice daily (35 µL drop -> 8.4 µCi, 119µg Brim) -> Cmax of radioactivity in choroid/retina Rabbits: twice daily 14 days 0.5% solution (35 µL drop -> 2 µCi, 113 µg Brim) -> Cmax of radioactivity in choroid/retina	Monkeys: 30.600 µg-Eq/g Rabbits: 20.8 µg-Eq/g	[95]
Betaxolol	Selektiver ß-Blocker	307.4 Da	1	2.81	Humans: 0.25% betaxolol twice daily for 28 days or longer -> 1290 ± 1170 ng/g in the choroid Monkeys: 0.25% betaxolol twice daily unilaterally for 30 days	Humans: 71.4 ± 41.8 ng/g Monkeys: 121 ng/g	[96]
Netardusil	ROCK-inhibitor	453 Da	1	4.73	Rabbits: single drop (35 µL of 14C-netarsudil 0.02% -> Cmax of in Retina-choroid	80 (left) or 50 (right) ng ∗ eq/g	[97]
Memantine (HCL)	Antagonist to nmDA-Receptors	179.2 Da (215.76 Da)	1	Memantine: 3.5 (hmdb.ca)	Arterially perfused bovine eye model: 4 mL of 9.27 mM memantine hydrochloride solution placed in reservoir on the eye (8.002 µg) -> Cmax retina 2046 ng/g vitreous 442 ng/g, Choroid/RPE 3894 ng/g after 9 h of perfusion	2046 ng/g	[98]
Drugs tested for AMD treatment							
Bevacizumab	recombinant humanized monoclonal antibody, inhibits VEGF-A	149 kDa	Negatively-charged at pH 7.4 [99]	Unknown (known to be lipophilic)	Pigmented rabbits: Bevacizumab eye drops (1.25 mg/0.05 mL six times daily for the first 7 days) -> 18.2 ± 4.2 ng/g in retina/choroid	18.2 ± 4.2 ng/g in retina/choroid	[100]
ESBA105	anti-TNF-alpha single-chain antibody fragment	26 kDa	-	-	Rabbits: 10 mg/mL ESBA105, 50 µL eyedrop -> 1 day hourly drops up to 10 h (up to 5 mg/day) -> Cmax: vitreous humor (295 ng/mL), neuroretina (214 ng/mL) and RPE-choroid (263 ng/mL) multi-day treatment: 9.6 mg/mL, 5 drops per day up to 6 days (up to 15 mg/6 days) -> Cmax: RPE-choroid (1298 ng/mL) vitreous humor (580 ng/mL) and neuroretina (917 ng/mL)	Single drop: 214 ng/mL Multi-day treatment: 917 ng/mL	[101]
Innovative small molecules							
GAL-101 (MRZ-99030)	Β-Amyloid aggregation modulator, dipeptide	289 Da		Computed logP-1.1	Monkeys: single eye drop -> >100 nM in the retina, via sclera and choroid	>100 nM	[102]
SF-0166	integrin αVβ3 antagonist	475.5 Da		Computed logP 2.7	Rabbits: Single eye drop of 50 µL 5% SF-0166 (2.5 mg/eye) -> Cmax retina-choroid 5103 ng/g	5103 ng/g in retina-choroid	[103]
Squalamine lactate	Inhibitor of VEGF, PDGF, and bFGF through intracellular mechanism	718.04 Da	2	Squalamine: 3.24	Laser-induced CNV rat model -> systemically administered squalamine lactate -> partially reduced choroidal neovascular membrane development No PK		[104]
Inhibitors of receptor tyrosine kinases							
TG100801, inactive prodrug of TG100572	inhibits Src kinases and selected receptor tyrosine kinases	580.1 Da (476)	1	7.64	Laser-induced CNV mouse model-> single 10 µL drop of 1% TG100801: Cmax (TG100801) -> 242 nM (retina), 1680 nM (Sclera/choroid); Cmax (TG100572) -> 97 nM (retina), 2460 nM (Sclera/Choroid); Dutch belted rabbits- > 1 40 µL drop of 0.6% TG100801: nach 2 h TG100801 -> 46 nM (retina), 34 nM (Choroid), TG100572 -> 41 nM (retina), 169 nM (choroid)	Mouse: TG100572 -> 97 nM Rabbit: TG100572 -> 41 nM	[105]
pazopanib	targets multiple receptor tyrosine kinases such as VEGF receptors	437.5 Da	0	3.55	Laser-induced CNV rat model -> twice daily topical eye drop treatment -> decreased leakage from photocoagulated lesions by 89.5% (*p* < 0.001); inhibited thickness of the developed CNV lesions by 71.7% (*p* < 0.001) No PK		[77]
Acrizanib (LHA510)	small-molecule VEGFR-2 inhibitor	445.40 Da	1	2.93	PK: brown Norway rats tid for 10 days (4 µL x 0.3% suspension) and 1 drop on day 11 -> Cmax 1910 nM (retina)	1910 nM	[106]
PAN-90806	VEGFR2 tyrosine kinase inhibitor	532.4 Da	0	Computed logP 3.7	Topical administration led to significant and sustained drug levels in retina and choroid, as well as suppression of neovascularization in various models		[107]

* predicted physiological charge and logP obtained from https://go.drugbank.com (accessed on 21 December 2021); computed logP obtained from https://pubchem.ncbi.nlm.nih.gov (accessed on 21 December 2021); in red: tested in clinical trials.

**Table 4:** Overview on drug delivery systems for topical delivery to the posterior segment.

Delivery System (Drug)	Size	Characteristics	Pharmacokinetics	Further Results	Ref.
Amino acid/Peptid-based drug delivery					
Valine-hemisuccinate ester prodrug: Val-HS (THC)	THC-Val-HS 513.6 Da; THC 314.2 Da	Higher aqueous solubility, higher polar surface area, improved logD (pH 7.4)	Rabbits: 2x daily for 5 days 50 µL THC-Val-HS in Tocrisolve emulsion (300 µg THC) -> THC-Val-HS: 15.5 ng/50 mg retina-choroid, THC: 5.2 ng/50 mg retina-choroid after 1 h	IOP-lowering equivalent to pilocarpine in a rabbit glaucoma model	[120]
Cell-penetrating peptide (CPP) HIV transactivator of transcription (TAT) (acidic fibroblast growth factor (aFGF))	Tat-aFGF-His: ~17.3-kDa	TAT is positively charged (11 amino acids: GRKKRRQRRRC) [121]	Rats: 40 µL drop (2 µg TAT-aFGF-His) -> His+ cells peaked after 30 min, still detectable after 8 h in the retina (mainly retinal ganglion cells)	Strong protection against ischemia-reperfusion injury in rats	[122]
CPP TAT (calpain inhibitory peptide) -> Tat-µCl	Tat-µCl: 2857.37 Da (23 amino acids)	TAT is positively charged (11 amino acids: GRKKRRQRRRC) [121]	Rats: 7 days twice daily (20 µL of 1 mM Tat-µCl) -> Cmax 15.3 pg/µg protein in the retina 1 h after last drop	Tat-µCl was diffusely distributed throughout the retina	[123]
CPP polyarginine-6 (bevacizumab)		(5[6]-carboxyfluorescein-RRRRRR-COOH)	Rat: single 20 µL eye drop of bevacizumab (25 µg/µL) -> Cmax 1.65 ± 0.26 in the retina after 40 min; Porcine eyes: 20 µL drop (25 µg/µL) -> 0.10 ± 0.03 µg per retina	Mouse model of CNV: CPP and bevacizumab eye drops (twice daily 5 µL for 10 days) significantly reduced CNV lesions, comparable to anti-VEGF injection	[124]
Lipid-based drug delivery					
Annexin V liposomes (bevacizumab)	Mean diameter of 163 nm	On interaction with PS containing membranes, annexin V is reported to form higher order structures that induce formation of actin-independent endocytic vesicles [125]	Rats: Single 30 µL drop (13 mg/mL Avastin) -> 127 ng/g in the posterior eye; Rabbit: 30 µL (25 mg/mL Avastin) once daily for 5 days -> 18 ng/g in retina/choroid		[126]
Annexin V liposomes (TGF-ß1)	Mean particle size 157 nm	Surface charge of liposomes became more negative with annexin V	Rabbits: 30 µL twice in 5 min (125 ng/mL TGF-ß1) -> Cmax 114.7 pg/mL in the vitreous		[127]
Solid lipid nanoparticles SLN (Indomethacin); Nanostructured lipid carriers NLC (indomethacin)	Particle size: IN-SLN 226 ± 5 nm IN-NLC 227 ± 11 nm	Colloidal nanoparticulate dispersions -> biocompatible and mucoadhesive	Rabbits: Two x 50 µL eye drops -> retinal-choroidal IN-concentrations of 227 ng/g with IN-SLN und 893 ng/g with IN-NLC	Improved transcorneal permeability and retention characteristics of IN	[128]
DNA-based drug delivery					
Lipid DNA-Nanoparticles (Brimonidine)	NP alone: 10 nm	Amphiphil, lipophilic core, and hydrophilic corona; Aptameric and hydrophobic drug loading		Higher IOP reduction than Briminodine alone in DBA/2J mice	[129]
Lipid DNA-Nanoparticles (Travoprost)	NP alone: 10 nm	Amphiphil, lipophilic core, and hydrophilic corona; Aptameric drug loading	Albino rats: single drop of Trav-NP or Trav (80 µM) -> travoprost after 1 h: 434.9 pg/mg (Trav-NP) compared to 230.3 pg/mg (Trav)		[130]
Carbohydrate-based drug delivery					
Sodium carprate and hydroxypropyl methylcellulose solution (28 kDa antibody fragment -> specificity for the rat CD4 molecule)	28 kDa	Solution with penetration enhancer 0.5% sodium caprate and viscosity enhancer 1.5% hydroxypropyl methylcellulose	Rabbits: 50 µL eye drops at 20 min intervals over 12 h -> 50–150 ng/mL in the vitreous		[131]
γ-cyclodextrin (CD) (dexamethasone) nanoparticle	100–300 nm drug/CD complexes CD: 1–2 kDa	Shaped like truncated cones, with a hydrophilic outer surface and a somewhat lipophilic central cavity	Rabbits: 1.5% dex- amethasone/γCD eye drops (50 µL) 3 doses in left eye for 15 days -> left eye: 201 ± 48 ng/g, right eye: 64 ± 12 ng/g in the retina		[132]
Chitosan oligosaccharide (CSO) nanomicelles (dexamethasone)	100 nm	CSO + Valylvaline (VV) + stearic acid (SA); VV is targeting PepT-1 -> faster crossing of conjunctival and scleral barriers	Rabbits: 3 × 50µL CSO-VV-SA -> at 0.5 and 1 h Dex conc. reached therapeutic levels (>200 ng/g) in sclera-choroid-retina	Higher ocular retention time compared with traditional eye drops	[133]
